# Data Science Education for Residents, Researchers, and Students in Psychiatry and Psychology: Program Development and Evaluation Study

**DOI:** 10.2196/75125

**Published:** 2026-01-16

**Authors:** Hayoung K Donnelly, David Mandell, Sy Hwang, Emily Schriver, Ugurcan Vurgun, Graydon Neill, Esha Patel, Megan E Reilly, Michael Steinberg, Amber Calloway, Robert Gallop, Maria A Oquendo, Gregory K Brown, Danielle L Mowery

**Affiliations:** 1 Department of Psychiatry, Perelman School of Medicine, University of Pennsylvania, Philadelphia, PA, United States; 2 Institute for Biomedical Informatics, Perelman School of Medicine, University of Pennsylvania, Philadelphia, PA, United States; 3 University of Pennsylvania Health System, University of Pennsylvania, Philadelphia, PA, United States; 4 PennDNA, Perelman School of Medicine, University of Pennsylvania, Philadelphia, PA, United States; 5 Department of Biostatistics, Epidemiology and Informatics, Perelman School of Medicine, University of Pennsylvania, 3700 Hamilton Walk, Philadelphia, PA, 19104, United States, +1 215 746-6677

**Keywords:** data science, AI literacy education, artificial intelligence, psychiatry education, suicide research, natural language processing, cloud computing

## Abstract

**Background:**

The use of artificial intelligence (AI) to analyze health care data has become common in behavioral health sciences. However, the lack of training opportunities for mental health professionals limits clinicians’ ability to adopt AI in clinical settings. AI education is essential for trainees, equipping them with the literacy needed to implement AI tools in practice, collaborate effectively with data scientists, and develop skills as interdisciplinary researchers with computing skills.

**Objective:**

As part of the Penn Innovation in Suicide Prevention Implementation Research Center, we developed, implemented, and evaluated a virtual workshop to educate psychiatry and psychology trainees on using AI for suicide prevention research.

**Methods:**

The workshop introduced trainees to natural language processing (NLP) concepts and Python coding skills using Jupyter notebooks within a secure Microsoft Azure Databricks cloud computing and analytics environment. We designed a 3-hour workshop that covered 4 key NLP topics: data characterization, data standardization, concept extraction, and statistical analysis. To demonstrate real-world applications, we processed chief complaints from electronic health records to compare the prevalence of suicide-related encounters across populations by race, ethnicity, and age. Training materials were developed based on standard NLP techniques and domain-specific tasks, such as preprocessing psychiatry-related acronyms. Two researchers drafted and demonstrated the code, incorporating feedback from the Methods Core of the Innovation in Suicide Prevention Implementation Research to refine the materials. To evaluate the effectiveness of the workshop, we used the Kirkpatrick program evaluation model, focusing on participants’ reactions (level 1) and learning outcomes (level 2). Confidence changes in knowledge and skills before and after the workshop were assessed using paired *t* tests, and open-ended questions were included to gather feedback for future improvements.

**Results:**

A total of 10 trainees participated in the workshop virtually, including residents, postdoctoral researchers, and graduate students from the psychiatry and psychology departments. The participants found the workshop helpful (mean 3.17 on a scale of 1‐4, SD 0.41). Their overall confidence in NLP knowledge significantly increased (*P*=.002) from 1.35 (SD 0.47) to 2.79 (SD 0.46). Confidence in coding abilities also improved significantly (*P*=.01), increasing from 1.33 (SD 0.60) to 2.25 (SD 0.42). Open-ended feedback suggested incorporating thematic analysis and exploring additional datasets for future workshops.

**Conclusions:**

This study illustrates the effectiveness of a tailored data science workshop for trainees in psychiatry and psychology, focusing on applying NLP techniques for suicide prevention research. The workshop significantly enhanced participants’ confidence in conducting data science research. Future workshops will cover additional topics of interest, such as working with large language models, thematic analysis, diverse datasets, and multifaceted outcomes. This includes examining how participants’ learning impacts their practice and research, as well as assessing knowledge and skills beyond self-reported confidence through methods such as case studies for deeper insights.

## Introduction

Artificial intelligence (AI) applications in behavioral medicine now span a wide range, including detecting at-risk populations, assisting clinicians in decision-making, and providing feedback to improve the quality of interventions [1]. Exposure to data science and AI education is crucial for medical trainees, as it prepares them to implement AI tools in their practice with the necessary literacy, collaborate effectively with data scientists, and explore careers as interdisciplinary researchers [2,3]. Medical trainees recognize the importance of AI and support its inclusion in their curriculum [4,5]; however, they also report low levels of knowledge and skills in addition to anxiety about using AI [5-7]. As a result, AI literacy among clinicians remains low, hindering the adoption of AI in clinical settings [8,9].

Self-learning methods, such as online articles and media resources, have been found to be the most common way for students to learn AI, with more than 70% relying on these approaches [2,10]. However, only 36% of students have exposure to formal AI training [2]. Previous studies have highlighted the need for guided and structured AI education tailored to health trainees, such as formal curricula and workshops [3,8]. Despite this need, barriers to implementing formal AI education persist, primarily due to limited time within an already demanding medical curriculum. A trainee-centered approach to structured learning opportunities may help reduce their burden. This includes integrating AI training into the existing didactic schedule [11] and adopting a human-centered design from recruitment to workshop facilitation.

Trainees in psychiatry and psychology may face even more limited opportunities for AI training tailored to their specialty and interests. Most existing AI education programs focus on general AI concepts and broad medical applications rather than addressing field-specific needs [3]. While some programs have been tailored for a specific domain such as radiology, a systematic review showed a lack of AI education programs specifically designed for psychiatry and psychology trainees [3]. Clinicians’ learning of data science should be directly applicable to their area of expertise, helping them generate ideas for their research applications. For instance, AI education programs for psychiatry and psychology trainees should be designed around mental health–related questions, data sources, and AI applications to ensure meaningful translation into their research and clinical practice.

One area to consider in AI education for psychiatry and psychology trainees is the application of AI in suicide prevention. Researchers are leveraging AI to identify patients at risk for suicidal thoughts and behaviors in clinical settings and the wider population [12]. Research in this space can scan electronic health records (EHRs) to identify elevated suicide risk, improve ED suicide screening, and decrease racial disparities in suicide [12]. This underscores the importance of becoming familiar with these technologies as future clinicians. In particular, analyzing text data and developing natural language processing (NLP)–based AI can deepen the understanding of the complexity of suicidal ideation and behaviors, allowing for the design of contextually sensitive suicide prevention strategies. For instance, clinical notes from EHR and NLP have demonstrated their strengths in enhancing suicide prevention strategies [13,14]. Developing AI education focused on suicide risk would offer trainees valuable insights into leveraging AI for both their research and practice.

Finally, developing and implementing AI education for medical trainees requires incorporating a structured evaluation process to assess its effectiveness. However, among 41 existing AI education development studies in medicine, only 5 included formal evaluation methods, such as structured surveys and assessments, whereas the remaining 36 studies solely described the education programs without measuring their impact [8]. Without rigorous evaluation, it is difficult to determine whether these programs effectively enhance knowledge and skills among trainees. One study specifically highlighted the importance of a competency-based curriculum in enhancing AI knowledge and skills, ultimately increasing the adoption of AI in clinical practice [8].

To address these gaps, we developed and implemented a 3-hour workshop to introduce psychiatry and psychology trainees to the applications of AI in suicide research. Specifically, we incorporated NLP to highlight the potential of leveraging clinical text data for psychiatry research and adopted a program evaluation framework to systematically assess the program’s efficacy.

## Methods

### Overview

We designed, disseminated, and evaluated a 3-hour training workshop to introduce psychiatry and psychology trainees to data science and AI. The workshop was conducted using Python Jupyter notebooks, Microsoft Azure Databricks, and Zoom (Zoom Communications). We began with a brief introduction and presurvey (5 min), followed by a Databricks setup session originally planned for 15 minutes. Although participants’ accounts and workspace access had been configured in advance, the setup process took approximately 30 minutes, which was 15 minutes longer than expected. A site reliability engineer was present to help manage the situation effectively; however, we needed to cancel the welcome speech by a psychiatry faculty member. The introductory remarks were intended to highlight the need for NLP research in suicide prevention. Once all participants’ environments were set up, we began the first phase of the hands-on coding practice, which focused on data characterization and preprocessing (1 h 20 min). We took a 10-minute break and then continued with the second phase, which covered concept extraction and statistical analysis (1 h 20 min). The session concluded with a wrap-up that included a postsurvey and office hour sign-up (10 min).

### Ethical Considerations

It was determined by the University of Pennsylvania Institutional Review Board that this project is a quality improvement initiative that does not meet the definition of human subjects’ research. Hence, further institutional review board review is not required. Before starting the survey, participants were informed that the data would be used to develop or enhance future workshops and that survey participation was voluntary. No identifiable information was collected from the survey, and participants did not receive any compensation.

### Computing Environment

To optimize our instruction time during the workshop, we required an environment preconfigured with the appropriate security for storing and analyzing deidentified clinical data, access to workshop materials, and the necessary software packages for processing data. We leveraged a secure cloud computing environment within Microsoft Azure and created a workspace designed to meet Health Insurance Portability and Accountability Act security requirements and provide controlled access to the study materials. Lessons were constructed in Jupyter notebooks using the Microsoft Azure Databricks analytics platform and written in Python that explained rationale and demonstrated the processing steps in context.

### Workshop Materials

The main workshop materials included practice data and prewritten code in Jupyter notebooks. The coding practice primarily focused on running prewritten code rather than writing new code, as the registration survey indicated that most participants had little to no experience with Python coding and NLP. However, we also offered participants the flexibility to modify the code during or after the workshop if they wished. Most importantly, we prioritized creating an interactive learning environment rather than delivering 1-directional instruction. For example, we regularly checked in with participants to assist with any issues they encountered during the coding practice, and 3 data scientists, in addition to the instructors, were available to provide support.

Specifically, the objective of the workshop materials was to demonstrate the application of data science, AI, and NLP to answer 3 biomedical research questions (RQs) using a dataset of chief complaints from the EHR:

- RQ1: How many chief complaints are related to suicidality?
- RQ2: Is the prevalence rate of suicidality statistically different between White and Black or African American populations?
- RQ3: Does the prevalence rate of suicidality increase with age?

To answer these questions, we organized the workshop materials to address 4 subtasks in data science and AI: data characterization, data standardization, concept extraction, and statistical analysis. For data characterization, we demonstrated how to generate frequency distributions, identify missing data, and handle outliers within the dataset. For data standardization, we applied general standardization techniques with Python function *str,* including addressing lexical variation with case reduction (eg, replacing “SUICIDE” with “suicide”). We used the WordNetLemmatizer for lemmatization to address term variation within the same concept. For instance, “suicide,” “suicidal,” and “suicidality” were standardized to “suicide.” We detected negation using *negex* (eg, “no suicide thought” was classified as “negated”). We also applied domain- and context-specific standardization. For example, in medical settings within the state of Pennsylvania, “302” refers to involuntary commitment for emergency evaluation and treatment, whereas “201” refers to voluntary commitment. These context-specific terms were replaced with descriptive and general terms (eg, “302” was replaced with “involuntary commitment”).

For concept extraction, we developed a dictionary containing suicide-related terms (eg, “suicide ideation” and “fatal self-harm behavior”) and applied them using regular expressions. Finally, for statistical analysis, we demonstrated how to calculate the prevalence rate of suicidality between demographic groups and apply a *t* test to determine if there is a significant difference in prevalence rates between the groups, namely White and Black or African American populations. We also demonstrated how to examine the predictive effect of age (independent variable) on suicidality (dependent variable) using logistic regression. Two researchers drafted and demonstrated the code, and feedback from the Methods Core of the Penn Innovation in Suicide Prevention Implementation Research Center was integrated to improve the materials. The Methods Core’s mission is to support the Penn Innovation in Suicide Prevention Implementation Research Center’s suicide prevention research by applying computational, implementation science, and human-centered design approaches. Their role in the development and dissemination of this workshop was to provide feedback from both data science and clinical perspectives, ensuring that the workshop met the needs of psychiatry and psychology trainees and was developed in a user-friendly format. This included reorganizing NLP tasks to distinguish between content that target audiences may already know (eg, mental health–related terms) and content they may not know (eg, NLP-specific language). Feedback also recommended introducing types of clinical data (eg, chief complaints), referencing existing studies related to the research problem (eg, health disparities), and designing psychological research questions (eg, predicting suicide risk by race, ethnicity, and age).

### Trainee-Centered Recruitment

Human-centered design was used to design the recruitment flyer, which prioritized clarity and appeal through the use of a logical visual hierarchy and accessible language and by highlighting speaker credentials to build trust and credibility. A QR code, which directed users to the Research Electronic Data Capture registration survey, was included in the flyer to simplify the registration process and minimize barriers to action in an effort to maximize participation. A digital flyer was shared via the Department of Psychiatry’s email listserv at the University of Pennsylvania to recruit workshop participants. We did not restrict the number of workshop participants. Participation was open to any trainees (ie, students, postdoctoral researchers, and residents) in the Departments of Psychology and Psychiatry. With the aim of reducing the burden on trainees participating in the data science workshop alongside their regular training requirements, we collaborated with the resident training office. To enhance dissemination efforts, we aligned the workshop schedule with the annual resident training calendar, leveraged prescheduled didactics time for trainees, and remained flexible toward other obligations. The 3-hour training workshop took place in August 2024 during a prescheduled weekday didactic session for trainees.

### Workshop Evaluation

**Figure 1.**
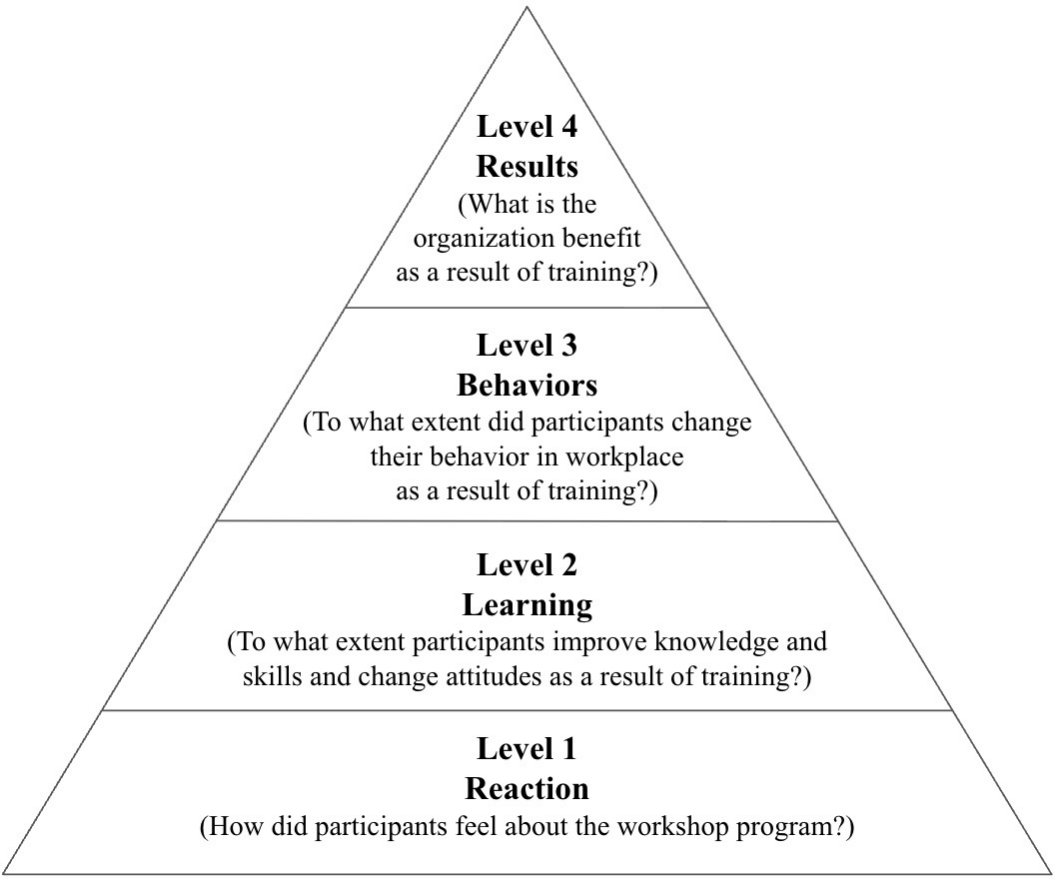
Kirkpatrick model for program evaluation [15].

We adopted the Kirkpatrick program evaluation model [15], a model for evaluating the impact of training programs (Figure 1). The Kirkpatrick program evaluation model entails 4 levels of evaluation: level 1 (reaction), level 2 (learning), level 3 (behaviors), and level 4 (results). Specifically, we evaluated the impacts of our learning modules for participating trainees using level 1 (reaction) and level 2 (learning). Level 1 (reaction) assesses whether trainees found the training helpful. Level 2 (learning) assesses whether the trainees acquired the intended knowledge and skills from the program.

At level 1, participants’ overall satisfaction was assessed using 2 questions: (1) “Overall, did you find the workshop helpful?” rated on a 4-point Likert scale, ranging from 1 (not helpful) to 4 (very helpful); and (2) “How likely are you to recommend this workshop to others?” rated on a 4-point Likert scale, ranging from 1 (very unlikely) to 4 (very likely). Participants were also asked to identify specific components of the training they found helpful, with options including the introduction to overall NLP concepts, coding demonstrations, and materials that were provided. Each component was rated as 1 (helpful) or 0 (not helpful).

At level 2, the confidence level of NLP knowledge and Python coding skills was measured with 8 questions (eg, “How confident are you in the following concepts or skills?”) with a 4-point Likert scale, ranging from 1 (not confident) to 4 (very confident). Specifically, confidence in NLP knowledge was assessed using 4 questions, each evaluating participants’ confidence in data characterization, data standardization, data transformation from unstructured to structured data, and concept extraction. Participant confidence in their Python coding skills was measured with 4 questions assessing participants’ confidence in performing tasks such as data standardization, data transformation, statistical analysis, and visualization using Python. We evaluated changes in confidence level before and after the workshop using paired *t* tests for the 6 participants with pre- and post-training data. Additionally, open-ended questions such as “Are there other topics you wish future workshops to cover?” were included to gain insights into ways to revise the workshop.

## Results

### Level 1: Reaction Evaluation

A total of 10 trainees participated in the workshop virtually. They included residents, postdoctoral researchers, and graduate students from the psychiatry and psychology departments. All participants completed the presurvey, whereas 6 (60%) participants completed the postsurvey. Only 2 (20%) of the 10 participants had experience with Python or NLP training before this workshop.

The participants found the workshop to be helpful overall (mean 3.17, SD 0.41) and expressed a willingness to recommend it to others (mean 3.17, SD 0.41). All participants found the coding demonstration and the learning materials helpful, and 5 (83%) of 6 participants found the introduction to NLP concepts helpful.

### Level 2: Learning Evaluation—NLP Knowledge

**Figure 2.**
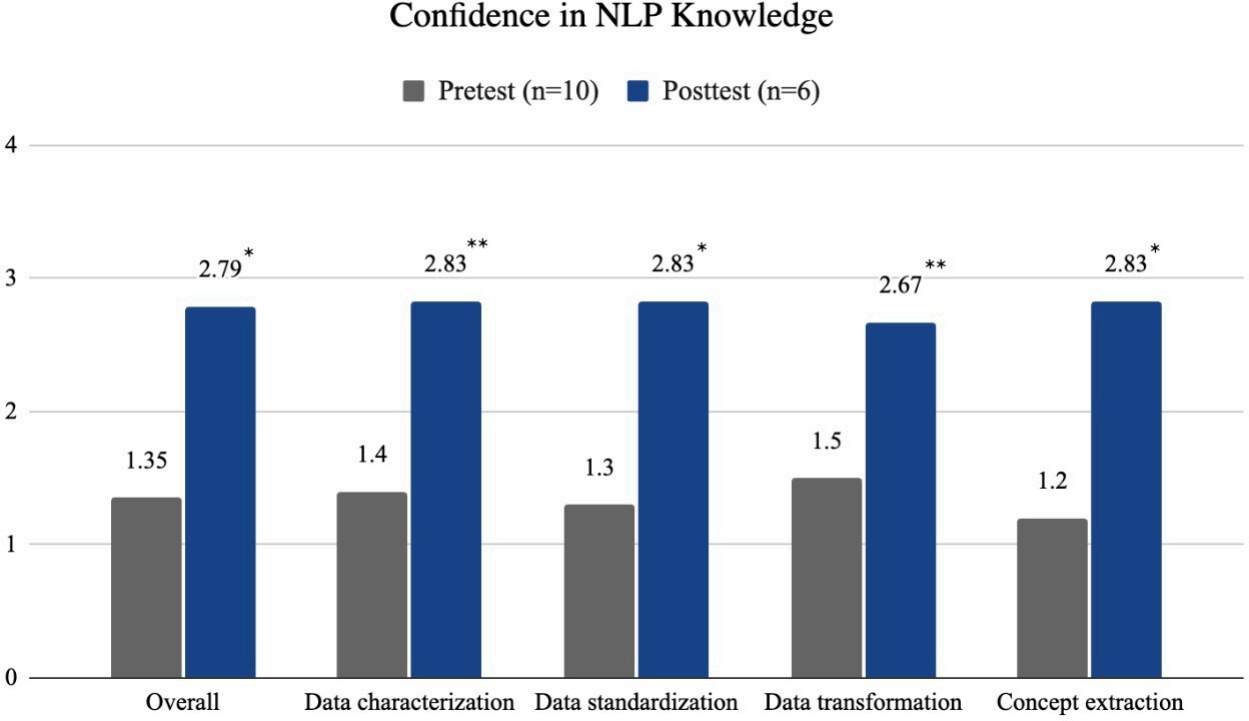
Confidence levels in natural language processing (NLP) knowledge before and after the workshop. *P<.05, **P<.01.

Participants’ confidence in their overall knowledge significantly increased (*P*=.002) from 1.35 (SD 0.47) before the workshop to 2.79 (SD 0.46) after the workshop (Figure 2). Participants’ confidence in each individual topic area also significantly improved. Participants’ confidence in characterizing clinical text data increased (*P*=.01) from 1.40 (SD 0.70) to 2.83 (SD 0.70). Confidence in text data preprocessing increased (*P*=.002) from 1.30 (SD 0.67) to 2.83 (SD 0.32). Confidence in transforming from unstructured to structured data improved (*P*=.01) from 1.50 (SD 0.53) to 2.67 (SD 0.42). Their confidence in extracting concepts from text data increased (*P*=.004) from 1.20 (SD 0.42) to 2.83 (SD 0.97).

### Level 2: Learning Evaluation—Python Coding Skills

**Figure 3.**
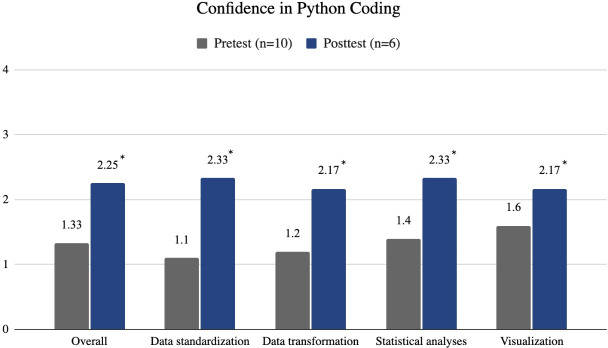
Confidence levels in python coding before and after the workshop. *P<.05.

Participants’ confidence in their overall coding abilities also significantly improved (*P*=.01), increasing from 1.33 (SD 0.60) to 2.25 (SD 0.42) as demonstrated in Figure 3. Participants reported significant improvements in confidence across all 4 individual Python coding skills. Confidence in standardizing text data increased (*P*=.01) from 1.10 (SD 0.75) to 2.33 (SD 0.52). Similarly, confidence in transforming unstructured data into structured data improved (*P*=.01) from 1.20 (SD 0.52) to 2.17 (SD 0.41). Confidence in conducting statistical analysis (ie, descriptive statistics) using Python increased significantly (*P*=.01) from 1.40 (SD 0.75) to 2.33 (SD 0.52). Confidence in visualizing analysis results increased significantly (*P*=.04) from 1.60 (SD 0.75) to 2.17 (SD 0.41).

In addition to the quantitative findings, qualitative feedback included the positive experiences of participants with the workshop. Overall, the feedback from the open-ended questions by trainees was positive and insightful (eg, “I’m glad you did this workshop, I hope there are more to come with some more hands-on experiences, but I think this served as a good introduction to the process and rationale behind the code.”). Feedback also included suggestions for incorporating thematic analysis and exploring additional datasets for future workshop development, which are discussed further in the following section.

## Discussion

### Principal Findings

This study illustrates the effectiveness of a tailored data science workshop for trainees in psychiatry and psychology, focusing on applying NLP techniques and clinical notes for suicide prevention research. The workshop significantly enhanced participants’ skills and confidence in conducting data science research, especially for those with substantial clinical experience but limited AI research training.

We received constructive comments from trainees through open-ended questions to guide future training efforts ranging from data-driven use cases, Python coding, and advanced NLP skills. In terms of data-driven use cases, trainees thought the workshop could be enhanced by “ ...work[ing] independently with data sets like national available data sets” and “starting our own projects working with real data.” We could incorporate more experiential training by providing more diverse datasets, including national datasets such as the National Longitudinal Study of Adolescent to Adult Health dataset or recommending that users bring their own dataset to apply these approaches. One trainee expressed an interest in “how to learn basics of coding - like any resources that would be helpful”; links to Python coding support could be provided or a Python coding bootcamp could be offered for trainees with less coding skills before the workshop. Another trainee recommended incorporating thematic analysis, for example, incorporating clustering techniques for deriving latent patterns in data. Additionally, future workshops could cover advanced NLP methods, including large language models (eg, GPT-4) to complete NLP subtasks. We could also incorporate exploring multifaceted outcomes, including clinical pathways, readmissions, and mortality, to demonstrate how to identify actionable insights from data.

### Limitations

Our study has several limitations. First, we implemented this study among trainees from a single institution. Feedback might vary if trainees had more or less experience with NLP and Python coding skills at another institution. Second, in terms of assessing learning, we measured confidence rather than actual knowledge or skill in level 2. We could also assess actual levels of knowledge and skills beyond self-reported confidence using methods such as case studies for deeper insights. Third, in the future, we intend to examine whether trainees acquired level 3 (behaviors) and level 4 (results) learning. We could assess how participants’ learning impacts their practice and research in the long term. This includes whether participants applied the gained knowledge and skills to their clinical practice, research engagement, and health care improvements in the long term [16]. For instance, it would be valuable to assess whether participants are more likely to adopt NLP-based clinical support tools after the workshop (level 3) and whether increased use of these tools enhances the efficacy of clinical workflows in health care systems at a systemic level (level 4). The knowledge and skills gained may also encourage participants to initiate independent research or engage in grant writing (level 3) to improve suicide prevention services over time (level 4). Fourth, our pilot study included a relatively small sample size. We aim to increase the size of the course by making it accessible to other trainee cohorts, including informatics, nursing, and engineering students with interest in health and AI. Finally, this study was an open trial without a controlled experimental approach. Adopting controlled methodologies, such as randomized controlled trials, will be beneficial for validating these findings in future studies.

### Conclusions

We successfully developed and implemented a 3-hour virtual workshop to educate psychiatry and psychology trainees on using data science and AI for suicide prevention research. Our preliminary results suggest the workshop enhanced participants’ knowledge and coding abilities, establishing a strong foundation for future advanced curriculum and learning objectives.

## Abbreviations

AI: artificial intelligence
EHR: electronic health record
NLP: natural language processing
RQ: research question
